# Subzero cell division, respiration, and genomic traits of cryophilic *Arthrobacter agilis* Ant-EH-1 isolated from cold-arid Antarctic mineral soils

**DOI:** 10.3389/fmicb.2025.1620620

**Published:** 2025-10-28

**Authors:** Claudia Wood, Elisse Magnuson, Ethan Harrop, Elizabeth Trembath-Reichert, Mary Beth Wilhelm, Jacqueline Goordial

**Affiliations:** ^1^School of Environmental Science, University of Guelph, Guelph, ON, Canada; ^2^Great Lakes Institute for Environmental Research, University of Windsor, Windsor, ON, Canada; ^3^School of Earth and Space Exploration, Arizona State University, Tempe, AZ, United States; ^4^Space Science & Astrobiology Division, NASA Ames Research Center, Moffett Field, CA, United States

**Keywords:** psychrophile, cryophile, extremophile, *Arthrobacter*, permafrost, cold-adaptation

## Abstract

*Arthrobacter* are commonly isolated from cold soil environments globally, including those that regularly reach sub-freezing temperatures, suggesting that *Arthrobacter* have significant potential for growth and activity under temperature and stress extremes. *Arthrobacter agilis* strain Ant-EH-1 was isolated from nutrient-poor, cold-arid mineral soils from Elephant Head, Antarctica and its growth and activity at sub-freezing temperatures were characterized in this study. We observed different optimal temperatures for cell division compared with aerobic heterotrophic respiration in *A. agilis* Ant-EH-1. Cell division was observed from at least −5 °C to 30 °C, with the optimal (fastest) growth rate occurring at 25 °C. Microbial respiration was measured from −5 °C to 30 °C with optimal (maximum CO_2_ produced) respiration occurring at 5 °C. Cold temperature optima of respiration compared with cell division could be indicative of adaptation to the cold and oligotrophic conditions of Elephant Head, where increased cell division under *in situ* conditions could lead to competition within the nutrient-poor soil matrix. The genome of *A. agilis* Ant-EH-1 was consistent with observations of cold-adapted activity and included genes related to cold stress, osmotic and oxidative stress, pigment biosynthesis, and potential scavenging of components from necromass. Microscopy revealed morphological differences in this isolate at sub-freezing temperatures, likely due to membrane or lipid modifications. Currently there are a limited number of organisms in culture that are capable of sub-zero growth, so characterisation of the growth and activity of subfreezing adapted microbiota is critical for understanding the ecology of Earth’s cryosphere, has broad biotechnological potential, and can also give insight into the limits for life on our planet or the potential for life on other cold planetary bodies.

## Introduction

*Arthrobacter* species have been isolated from a broad variety of environments including soil (Lee et al., 2003), food (Irlinger et al., 2005), paintings (Heyrman et al., 2005), human clinical specimens (Mages et al., 2008; Funke et al., 1998; Hou et al., 1998), sea water (Chen et al., 2009), air (Li et al., 2004), ice (Liu et al., 2018; Kumar et al., 2015; Margesin et al., 2004), glacier cryoconites (Margesin et al., 2012), sub-glacial lakes (Singh et al., 2016), and Antarctic marine and lake sediment (Pindi et al., 2010; Chen et al., 2005; Reddy et al., 2000; Han et al., 2021). They are frequently isolated from extreme environments including the Antarctic and have a well-documented tolerance to cold temperatures globally (Pindi et al., 2010; Reddy et al., 2000; Dsouza et al., 2015; Gupta et al., 2004; Junge et al., 1998; Wang et al., 2009; Reddy, 2002; Cho et al., 2019; Vodickova et al., 2022; Mukhia et al., 2021). Elephant Head, located in Ellsworth Land, Antarctica, contains dry, ice-free soils and year-round sub-zero temperatures. Previous microbial activity (acetate mineralization) assays on soils from Elephant Head demonstrated that some, but not all, soils contained microbiota that could be active at the sub-zero conditions experienced *in situ* (Wood et al., 2024). Twenty-one bacterial isolates were previously cultivated from dry permafrost soils at Elephant Head as described in (Wood et al., 2024), with *Arthrobacter* the most prevalent genus, comprising seven of the 21 cultivated isolates. In this follow-up study, in order to determine whether these cultivated organisms are genetically adapted to the cold, and capable of activity *in situ* in the extreme Elephant Head environment, one *Arthrobacter* isolate capable of sub-zero growth (−5 °C) was chosen for further characterization of its cold adaptive capabilities and genomic traits.

## Methods

### Isolation and characterization

*Arthrobacter agilis* strain Ant-EH-1 was isolated from cold, dry surface soils collected from Elephant Head, Ellsworth Land, Antarctica (79°49.106’S 83°18.139 W). The average summer atmospheric temperature in Elephant Head is −10.3 °C, with a yearly average of −20.3 °C (McKay et al., 2019). Surface soils where *A. agilis* Ant-EH-1 was isolated from (“Site 1,” 0–10 cm depth), warm above 0 °C for only a few hundred hours during the year (an estimated ~500 h based on “Site 3” located 0.3 km away). Moisture content of the soils is less than 0.5%. Total organic carbon and nitrogen content is low (<0.07 and 0.007%, respectively; Wood et al., 2024).

Dry soil from Elephant Head was added to 1.5 mL of liquid media Reasoner’s 2A broth (R2B), incubated for 1 week at 15 °C, and then spread plated onto Reasoner’s 2A agar (R2A). The plate was incubated at 15 °C for an additional week. Pink-coloured colonies of *A. agilis* Ant-EH-1 were streaked for isolation and growth was characterized on R2A agar and in R2B liquid media at −10, −5, 0, 5, 15, 25, 30, and 37 °C. Growth in liquid media was measured via optical density (OD) at 600 nm using a spectrophotometer. Growth rate was calculated as the change in OD over time during the exponential phase of growth. Growth was also characterized on half strength and 1/10th strength R2A as well as on R2A agar plates amended with NaCl (5, 8, and 10%) at 15 °C to examine the salt tolerance of the isolate.

### Acetate mineralization radiorespiration assay

Acetate mineralization by isolate *A. agilis* Ant-EH-1 was evaluated by a radiorespiration assay using radiolabeled acetate (1,2-^14^C) as a carbon substrate. Microcosms were set up in 20 mL serum vials in triplicate with triplicate autoclaved negative controls. Each 5 mL volume microcosm contained: 4860 uL R2B media; 20 uL 1,2-^14^C acetic acid (0.043 μCi (~95,000 disintegrations per minute, dpm)); 20 μL of unlabelled acetic acid (3.75 M); and 100 uL of 5.45×106 CFU/mL of *A. agilis* Ant-EH-1 liquid culture in R2B. Each microcosm also contained a vial of 0.5 mL 1 M potassium hydroxide (KOH) as a carbon dioxide trap. Sterile media (100 μL R2B) was added to negative controls to give the same final volume to all incubations. Microcosms were incubated at 30, 25, 15, 5, 0, and −5 °C. Measurements of KOH radioactivity (correlating with CO_2_ released) were taken periodically by liquid scintillation spectrometry on a Beckman Coulter (CA, USA) LS 6000SC and percent mineralization calculated as in Wood et al. (2024) and Goordial et al. (2016a).

### DNA extraction and sequencing

*Arthrobacter agilis* Ant-EH-1 was grown on R2A at 15 °C for 1 week. Isolated colonies were suspended in 750 μL of Powerbead solution from the Qiagen DNeasy PowerLyzer PowerSoil kit and DNA extraction followed manufacturers protocol. DNA was eluted with 100 μL of DNAse-free water. Library preparation was completed using the Oxford Nanopore Rapid Sequencing Kit (SQK-RAD004) for use with the flongle flow cell following manufacturers protocol (Oxford Nanopore Technologies). *A. agilis* Ant-EH-1 DNA was loaded into three flongle flow cells and three replicate 24-h sequencing runs were carried out. High accuracy base calling was used for the first run and fast base calling was used for subsequent runs.

### Genome analysis for adaptive traits

Sequence data from three sequencing reactions were concatenated together for assembly and analysis. Assembly was performed using Canu (v 2.2) (Koren et al., 2017). Genes were annotated using Prokka (v 1.14.6) (Seemann, 2014) and GhostKOALA (Kanehisa et al., 2016). Completeness of metabolic pathways was visualized with KEGG Decoder (Graham et al., 2018). Additional gene prediction and functional annotation was performed within the Integrated Microbial Genomes (IMG) platform developed by the Joint Genome Institute, Walnut Creek, CA, USA (Markowitz et al., 2009). The complete genome sequence of strain *A. agilis* Ant-EH-1 is available for public access on the Joint Genome Institute Integrated (JGI) Microbial Genomes & Microbiomes (IMG) under Gold Study ID: Gs0160646. The 16S rRNA gene was amplified using PCR (primers 27F – 5’-AGAGTTTGATCCTGGCTCAG-3′ and 1492R – 5’-TACGGYTACCTTGTTACGACTT – 3′) and Sanger sequenced at The Centre for Applied Genomics, Toronto, Canada. The Classifier tool of the Ribosomal Database Project (RDPII) v 2.13 (Wang et al., 2007) and the NCBI GenBank database were used to identify the isolate. The sequence can be found on NCBI GenBank under accession number OQ383637.

### Scanning electron microscopy

Three *A. agilis* Ant-EH-1 liquid cultures grown in R2B were harvested for visualization with scanning electron microscopy (SEM): (1) growth at −5 °C for 16 months with visible flocculation (OD_600_ = 1.13); (2) growth at 5 °C for 16 months with no flocculation (OD_600_ = 1.57); (3) growth at 25 °C for 1 month with flocculation (OD_600_ = 1.4). Cultures were grown at −5 °C and 5 °C for extended periods to reflect the incubation period of the acetate mineralization assay and obtain sufficient biomass for SEM. Cultures were diluted in R2B to an OD_600_ of 1.0, and 1.5 mL of each diluted culture was centrifuged at 4,000 rpm for 4 min. Cultures without flocculation did not produce a visible pellet after initial centrifugation and were additionally centrifuged at 10,000 rpm for 4 min. Harvested cells were washed twice in phosphate buffer (35 mM K_2_HPO_4_ and NaH_2_PO_4_) with centrifugation for 4 min at 4,000 rpm for cultures with flocculation and 10,000 rpm for cultures without flocculation. Washed pellets were resuspended in 400 μL of phosphate buffer, of which 200 μL was placed on a carbon planchet and incubated for 30 min to allow cell adhesion. After adhesion, planchets were gently submerged in 0.075% ruthenium red and 2.5% glutaraldehyde in 100 mM HEPES pH 7.3 for 30 min to fix cells, then washed once in 100 mM HEPES pH 7.3 and twice in MilliQ water. Cells were then dehydrated by submerging the planchets sequentially in 50, 70, 80, 90, 100%, and a second round of 100% ethanol for 10 min each, followed by drying with a Denton DCP-1 Critical Point Dryer (NJ, USA). Planchets were then mounted on pin stubs with double-sided carbon tape and coated in gold with a Denton Desk V TSC sputter coater. Cells were imaged on a FEI Quanta FEG 250 SEM (OR, USA) at the University of Guelph Molecular and Cellular Imaging Facility (ON, CA).

## Results and discussion

### Growth and activity characteristics of *Arthrobacter agilis* Ant-EH-1

**Figure 1:**
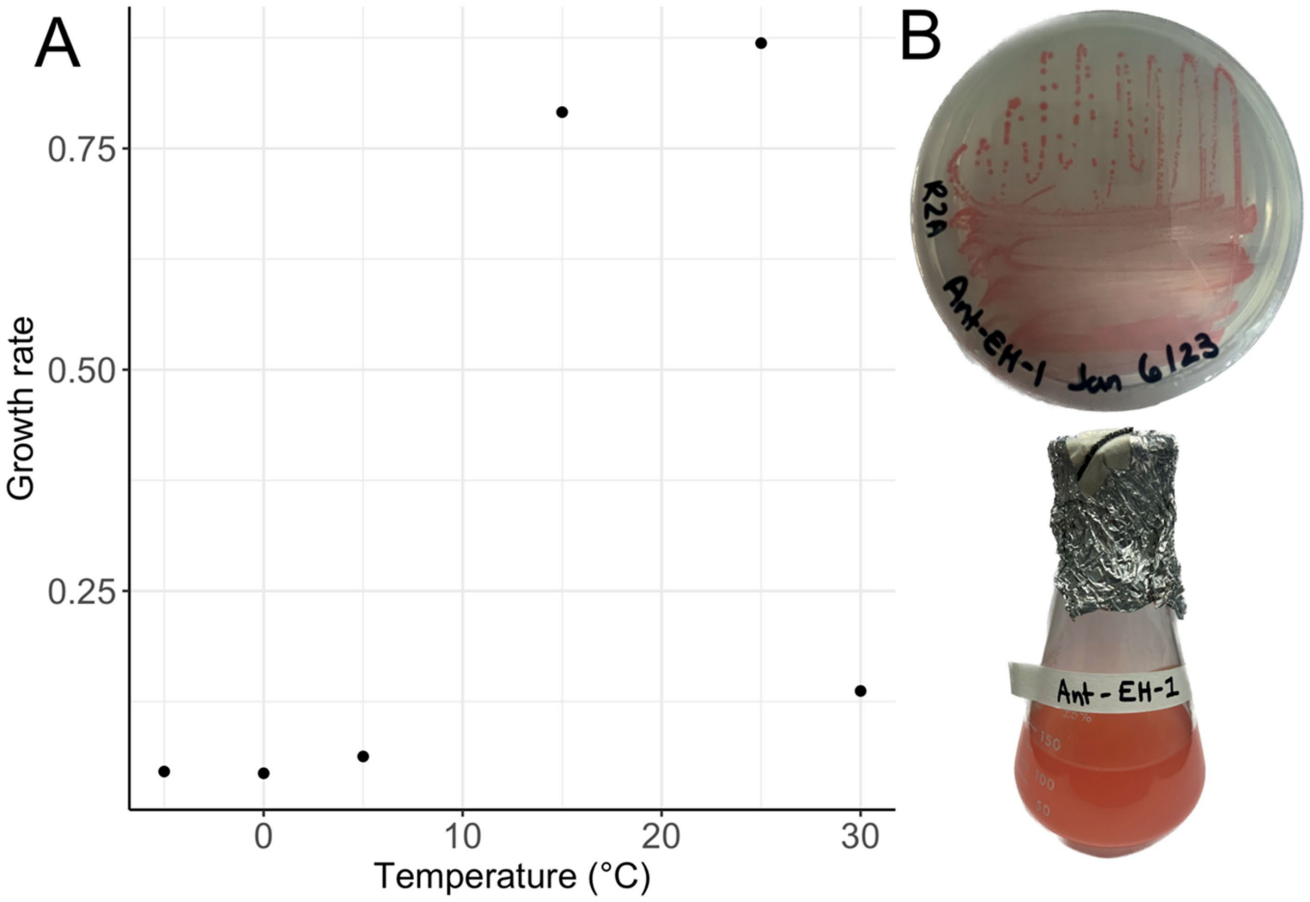
**(A)** Growth rate (h^−1^) of *Arthrobacter agilis* strain Ant-EH-1 in R2A from 30 °C to −5 °C based on optical density measurements (600 nm). **(B)** *Arthrobacter agilis* strain Ant-EH-1 colonies grown on R2A plate (top) and in liquid R2B (bottom).

*Arthrobacter agilis* strain Ant-EH-1 grew on R2A media forming bright pink, circular colonies on plates, and pink colouration in liquid R2B media (Figure 1) and was capable of growth from −5 °C to 30 °C on solid and liquid media. Optimum (fastest) growth rate based on optical density occurred at 25 °C (Figure 1) in R2B media, a common nutrient media for growth of oligotrophic organisms. This optimum temperature is similar to other *Arthrobacter* isolates from polar environments (Supplementary file 1). *A. agilis* Ant-EH-1 was capable of growth at lower nutrient concentrations (two and ten-fold diluted R2A), and maintained its viability in culture after 1 year (e.g., could be re-streaked from R2A plates incubated at −5 °C for 1 year) indicating its ability to survive long periods of time at sub-zero temperatures without the input of new nutrients. *A. agilis* Ant-EH-1 was previously observed to be halotolerant, capable of growth in media supplemented with up to 8% NaCl (Wood et al., 2024), like many other cryophilic organisms capable of sub-zero growth (Goordial, 2021). Halotolerance is thought to be needed by microbiota for activity and survival in permafrost, as solutes are concentrated into brine veins at subfreezing temperatures.

**Figure 2:**
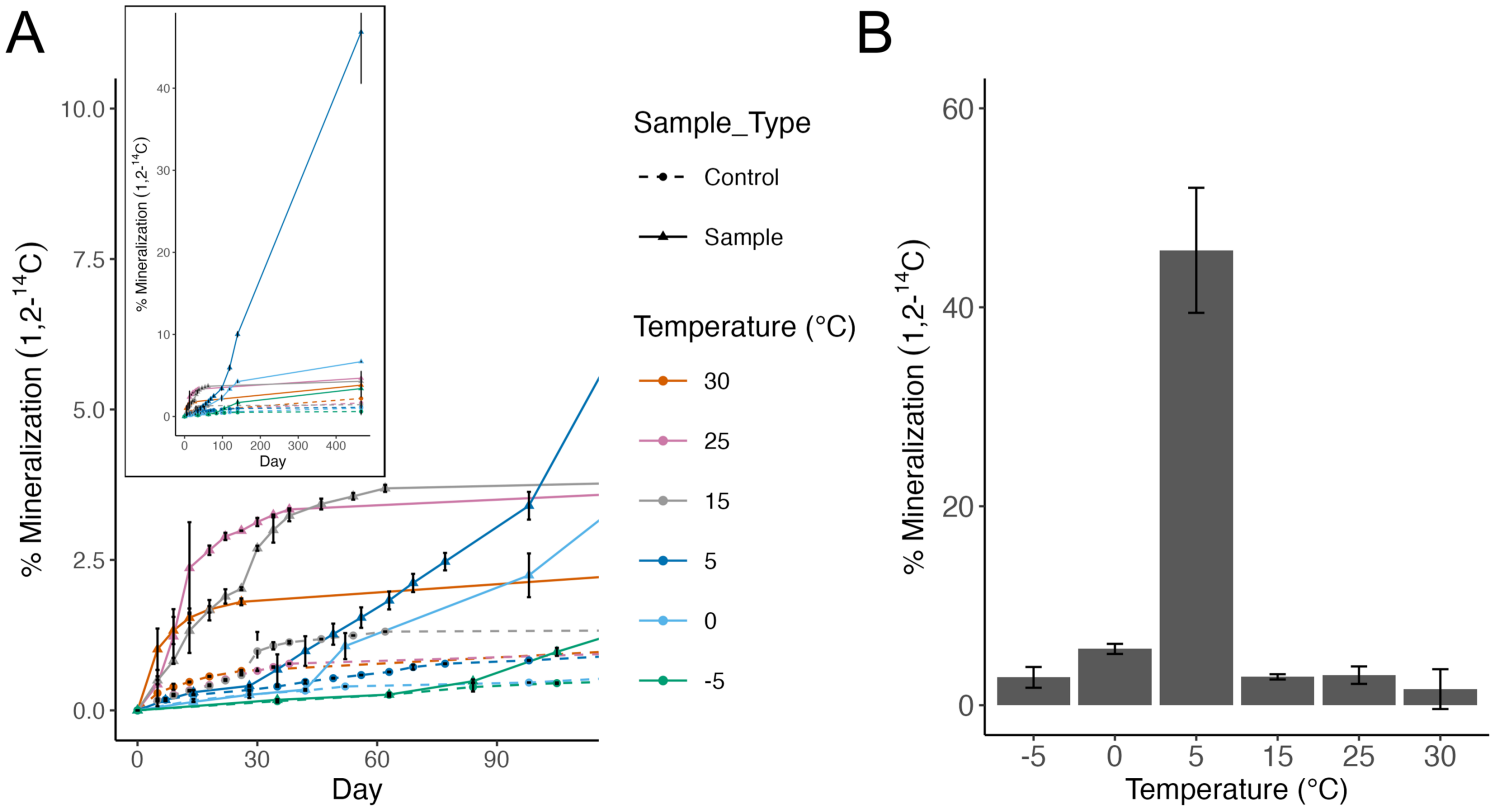
**(A)** Microbial activity assessed by the mineralization of radiolabeled acetate (1,2-^14^C) to carbon dioxide (CO_2_) by *Arthrobacter agilis* strain Ant-EH-1. Measurements are cumulative. Dashed lines show negative controls (sterile R2A media). Error bars show standard deviation of triplicate incubations. Inset shows activity to day 466. **(B)** Cumulative percent mineralization of radiolabeled acetate to carbon dioxide by *A. agilis* Ant-EH-1 after 466 days. Background levels in negative controls were subtracted from corresponding samples.

Heterotrophic respiration of acetate was detected at all temperatures tested from −5 °C to 30 °C (Figure 2A). By day 100 of the incubation, 5 °C surpassed all warmer temperatures tested in total percent acetate mineralized. Temperatures below 5 °C demonstrated lag phases that were 50–80 days in length compared with warmer temperatures which had short to no lag phase observed. Total acetate respired in incubations at warmer temperatures (>5 °C) plateaued quickly (day 15–40) compared to 5 °C which did not plateau by the end of the experiment after 466 days (Figure 2A), inset. The highest cumulative percent mineralization of acetate occurred at 5 °C (45%), compared with all other temperatures tested (Figure 2B). The second highest acetate mineralization was observed at 0 °C with 5.6% mineralization.

*Arthrobacter* genera have been cultivated from several Antarctic environments, and many are capable of growth at cold temperatures (0 to 5 °C) (Pindi et al., 2010; Chen et al., 2005; Reddy et al., 2000; Dsouza et al., 2015; Gupta et al., 2004; Wang et al., 2009; Reddy, 2002; Vodickova et al., 2022; Fong et al., 2001; Aislabie et al., 2013; Shen et al., 2021). *Arthrobacter agilis* Ant-EH-1 was closely related (similarity based on 16S rRNA gene) to other *Arthrobacter* from cold environments, including Antarctica (Supplementary Figure S2). To the best of our knowledge this is the first time that cell division has been documented via optical density for an *Arthrobacter* species at sub-zero temperatures as low as −5 °C. Prior studies stopped cultivation attempts at 4, 0 °C or −1 °C (Supplementary file 1), thus it is possible these isolates could be capable of cell division at lower temperatures. One prior study confirmed that an *Arthrobacter* species was capable of activity (measured via carbon dioxide production) down to −17 °C (Panikov and Sizova, 2006). *A. agilis* Ant-EH-1 had a maximum growth rate at 25 °C based on optical density measurements but showed significantly higher levels of activity at 5 °C based on radiorespiration assays. *A. agilis* Ant-EH-1 could be classified as a eurypsychrophile based on its ability to grow from −5 °C to 30 °C with a maximum growth rate above 20 °C (Raymond-Bouchard et al., 2018; Cavicchioli, 2016). Eurypsychrophiles can grow across a broad range of temperatures (temperature max > 30 °C, min below 0 °C) and have optimum growth rates around 20 °C (Raymond-Bouchard et al., 2018; Cavicchioli, 2016). However, fast growth rate at warmer temperatures does not necessarily indicate that these conditions are preferred by *A. agilis* Ant-EH-1. As suggested by Cavicchioli (2016) fast growth is not always better, especially in oligotrophic conditions such as those that exist in Elephant Head soils. Differences in activity and OD measurements suggests that *A. agilis* Ant-EH-1 employs different growth strategies at different temperatures. At warm temperatures it shows a rapid increase in activity and OD which plateaus quickly. At colder temperatures *A. agilis* Ant-EH-1 shows a slower initial increase in activity but then is able to sustain an active population for longer. Future transcriptomic analysis could help to determine if there is a different growth strategy employed at cold temperatures versus the slowed rates being a result of slowed kinetic reactions. This was demonstrated previously in transcriptomic studies of *Psychrobacter* sp. which found that it shifted from a fast-growing state at warmer temperatures (6–22 °C) to a resource efficiency state at cold temperatures (<4 °C) via downregulation of genes involved in energy metabolism (e.g., electron transport chain, TCA cycle) and biosynthesis (e.g., amino acid, nucleotide, ribosome, peptidoglycan synthesis) and upregulation of RNases and peptidases indicating a growth control response (Bergholz et al., 2009).

### Cell envelope characteristics of *A. agilis* Ant-EH-1 across a temperature gradient

**Figure 3:**
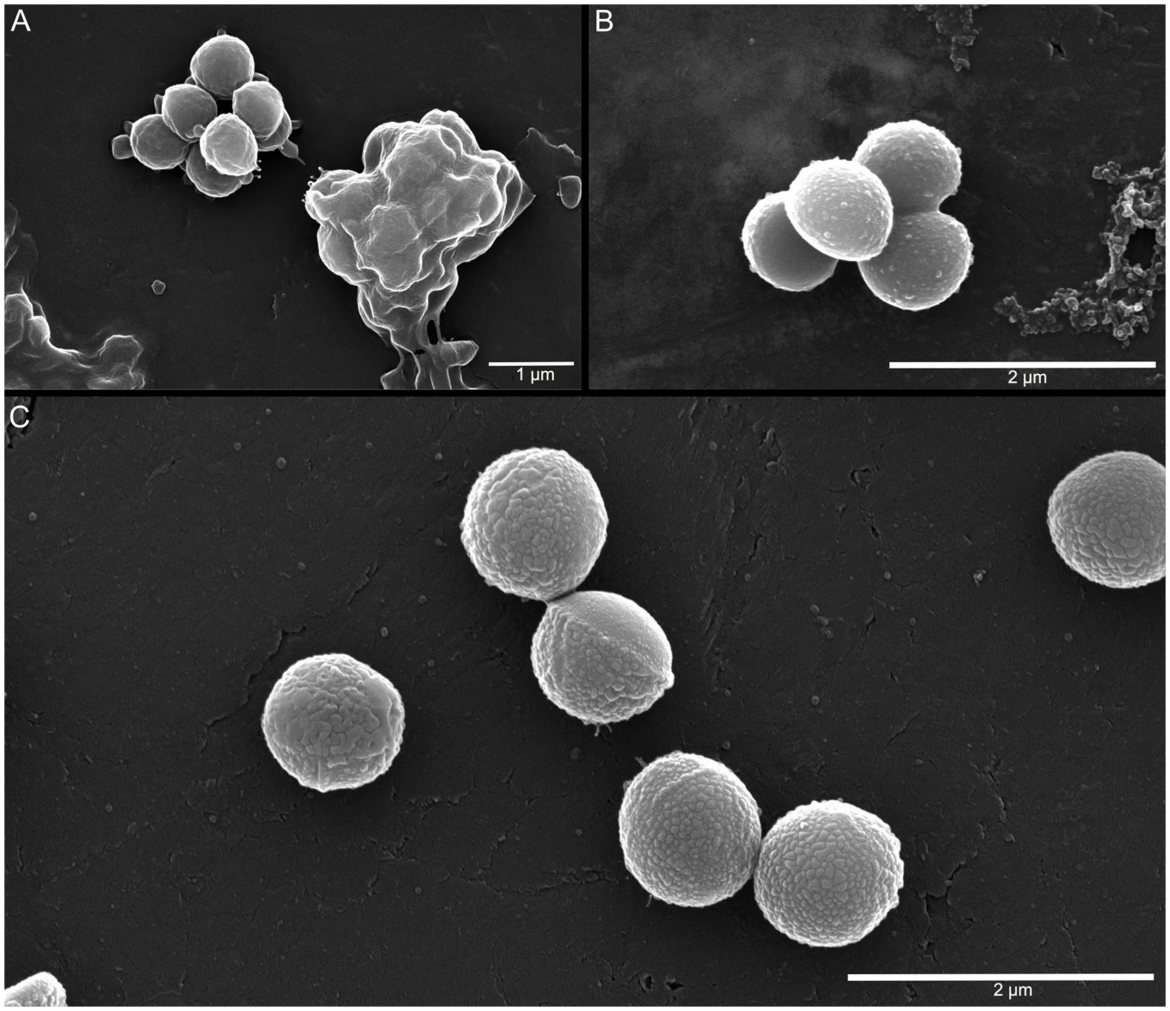
SEM images of *A. agilis* Ant-EH-1 grown at **(A)** 25 °C; **(B)** 5 °C; **(C)** −5 °C.

As the *A. agilis* Ant-EH-1 genome contained genes associated with cold-adaptive membrane and cell wall modifications, cultures grown at −5, 5, and 25 °C were visualized with SEM to identify potential temperature-dependent changes to the cell envelope. At all temperatures, cultures contained aggregated cells coated in extracellular polymeric substance (EPS)-like material (Figure 3). EPS have an array of functions in mediating cell--environment interactions, including formation of biofilms and aggregates and protection against environmental stressors (Costa et al., 2018). They are proposed to act as a cryoprotectant by limiting ice crystal formation and lowering the freezing point of water, among other potential mechanisms (De Maayer et al., 2014). Elevated EPS production at low and sub-zero temperatures has been observed in psychrophilic and psychrotolerant bacteria (Marx et al., 2009; Caruso et al., 2018), including *A. agilis* strain L77, a psychrotroph isolated from a subglacial lake (Singh et al., 2016).

Cells grown at 5 °C and −5 °C had nodule-like features partially (5 °C) or completely (−5 °C) covering the cell envelope (Figure 3), which may indicate cell wall modifications associated with cold adaptation. For example, dense nodular encrustations were observed in the psychrophile *Planococcus halocryophilus* Or1 grown at −15 °C as a result of peptidoglycan accumulation and calcium carbonate biomineralization (Mykytczuk et al., 2016). The genes associated with these accumulations in *P. halocryophilus,* peptidoglycan synthase (*ftsI*) and carbonic anhydrase (*cab*), were both identified in the *A. agilis* Ant-EH-1 genome. While the prominence of these nodules at −5 °C compared to 5 °C suggests a role in cold adaptation, it is unconfirmed whether these features were exclusive to *A. agilis* Ant-EH-1 grown at cold temperatures as thick EPS matrices at 25 °C may have obscured surface features. Additional transcriptomic and microscopic analyses are warranted to further investigate potential cold adaptive strategies.

### General genome characteristics and cold adaptive and stress response genes in *Arthrobacter agilis* Ant-EH-1

The *Arthrobacter agilis* Ant-EH-1 complete draft genome is 3,764,186 bp in length with 68.04% GC content. There were 5,935 protein encoding genes predicted, of which 3,236 were assigned a predicted function and the remaining annotated as hypothetical proteins (Table 1). Consistent with other psychrophiles and cryophiles the organism had genomic traits associated with cold adaptation, stress response, and DNA repair. While many of the genes and pathways are found in non-extremophilic organisms as well, these traits have been identified as being important in cryophiles and they are often present in multiple copies, or with redundant pathways as described below.

**Table 1:** Genome features of *Arthrobacter agilis* strain Ant-EH-1.

Attribute	Value
Genome size (base pairs)	3,764,186
Protein coding bases	3,163,122 (84.03%)
G + C content	2,561,211 (68.04%)
Scaffold count	6
No. of genes	6,015
Total predicted protein coding genes	5,935
rRNA genes	11
16S rRNA	3
tRNA genes	46
Protein coding genes with predicted function	3,236
Protein coding genes without predicted function	2,699

#### Cold and stress response

The *Arthrobacter agilis* Ant-EH-1 genome contains genes that facilitate general stress response (universal stress proteins, Kvint et al., 2003) as well as genes that facilitate cold adaptation via osmotic tolerance, oxidative stress, membrane and cell wall alterations, carotenoid biosynthesis, and DNA repair (Table 2; Supplementary materials). Coding regions for cold shock protein (*cspA*) were present in the genome. *CspA* can act as RNA chaperones and help with unfolding misfolded proteins and preventing misfolding during and after cold shock (Gottesman, 2018; Zhang and Gross, 2021). *A. agilis* Ant-EH-1 also has coding sequences for a variety of other chaperone proteins which can assist with protein folding under stressful conditions (*clpB, dnaJ, dnaK*) (Alam et al., 2021; Maillot et al., 2019).

**Table 2:** Cold adaptation and stress response genes present in the *Arthrobacter agilis* strain Ant-EH-1 genome.

Gene	Number of CDS^a^
Cold shock and general stress	
Cold Shock Protein (*cspA*)	3
Universal stress protein	10
Chaperone proteins (*clpB, dnaJ, dnaK, surA*)	3, 2, 4, 1
SOS-Response Transcriptional Repressor (*lexA*)	2
Osmotic stress	
Sodium/proton antiporter related genes (*nhaA, subunit A, subunit C, subunit D, nhaG*)	2, 3, 1, 1, 2
Potassium/proton antiporter related genes (*subunit khtT, yhaU, nhaP2*)	1, 3, 2
Osmoprotectant import ATP-binding protein (*osmV*)	2
Glycine Betaine ABC-Type Transporter (*betL, opuD, opuCB, gbuA*)	1,1,1,1
Proline/betaine transporter	2
Osmoregulated proline transporter (*opuE*)	2
Oxidative stress	
Superoxide dismutase [Mn]	2
Catalase	3
Lipoyl- Dependent Peroxiredoxin (*osmC*)	1
Putative Peroxiredoxin	2
Glutaredoxin-Like Protein (*nrdH*)	1
Thioredoxin 1 (*trxA*)	6
Thioredoxin Reductase (*trxR*)	2
Peptide Methionine Sulfoxide Reductase (*msrA/msrB*)	2
Mycothiol Acetyltransferase	4
Organic hydroperoxide resistance related genes	2
Membrane/cell wall alterations	
3-Oxoacyl-[Acyl-Carrier-Protein] Synthase I, II, III	2, 5, 1
3-oxoacyl-[acyl-carrier-protein] reductase (*fabG*)	8
All-trans-phytoene synthase/15-cis-phytoene synthase	2
Carotenoid Biosynthesis	
15-cis-phytoene synthase	2
Bisanhydrobacterioruberin hydratase	1
Dolichol-phosphate mannosyltransferase	1
DNA repair	
DNA Replication and Repair Protein (*recN, recF, recO*)	4, 2, 2
Excinuclease ABC Subunit A, B, C (*uvrA, uvrB, uvrC*)	7, 2, 3
ATP-Dependent DNA Helicase *uvrD/pcrA*	9
Deoxyribodipyrimidine Photo-Lyase (*phr, phr1*)	1
A/G - Specific Adenine Glycosylase (*mutY*)	1
Formamidopyrimidine-DNA Glycosylase (*mutM, fpg*)	1
DNA-3-Methyladenine Glycosylase I (*tagI*)	1
Methylated-DNA-[Protein]-Cysteine S-Methyltransferase (*ogt*)	2

a CDS, coding sequences.

In dry permafrost environments, water activity is low because aridity and freezing temperatures maintain any water present as vapour or ice. Liquid water present under these conditions may be due to porosity and mineral substrate composition, as well as the presence of solutes. Solutes will become concentrated in liquid water films and pockets present at sub-zero temperatures in the soil matrix, resulting in high solute concentrations which bind to water molecules and further reduce water availability for use by microorganisms (Devoie et al., 2024). One of the most well understood adaptations to osmotic stress is the accumulation of compatible solutes, or small organic molecules within the cell which help to maintain turgor pressure, depress the freezing point of intracellular water to prevent ice crystal formation, and prevent protein aggregation (Chattopadhyay, 2002; Chin et al., 2010; Roberts, 2005). Genes for the import or synthesis of compatible solutes glycine betaine, proline, and trehalose were present (glycine betaine transport related genes [*betL*, *opuD*, *opuCB*, *gbuA*), proline/betaine transporter (*opuAC*), trehalose synthase (*treS*, *treZ*, *treY*)] (Table 2). Inorganic cations including sodium (Na^+^) and potassium (K^+^) ions can become toxic to the cell if accumulated to high concentrations. *A. agilis* Ant-EH-1 contains genes for Na+/K + antiporters (*nhaA, nhaP, nhaD, mnhABCDEF*) which transport Na + and K + out of the cell and uptake H^+^ in order to maintain intracellular pH and cell volume while avoiding cytotoxic Na^+^/K^+^ accumulation (Vimont and Berche, 2000; Bremer and Krämer, 2019; Padan and Schuldiner, 1994; Padan and Schuldiner, 1994).

### Metabolism and adaptations for growth in oligotrophic conditions

#### Carbon utilization and storage

*Arthrobacter agilis* Ant-EH-1 carries out aerobic heterotrophic growth. Its genome encodes for complete glycolysis, pyruvate oxidation, tricarboxylic acid (TCA) cycle, and oxidative phosphorylation pathways, as well as a near-complete pentose phosphate cycle pathway (1 enzyme missing, transaldolase) (Table 3), though as this is a draft genome it is unclear whether this pathway is truly incomplete or instead reflects genome incompleteness. The *A. agilis* Ant-EH-1 genome also encodes for a complete glyoxylate shunt pathway (isocitrate lyase, *AceA;* and malate synthase, *GlcB*). The glyoxylate shunt bypasses the release of carbon dioxide (CO_2_) during the TCA cycle and may help conserve carbon in limiting environments. It was previously also identified in a bacterial isolate from nutrient-poor dry permafrost soils in University Valley, Antarctica where it was thought to help with carbon conservation in ~150,000-year-old permafrost (Goordial et al., 2016b).

**Table 3:** Nutrient stress genes present in the *Arthrobacter agilis* strain Ant-EH-1 genome.

Gene	Number of CDS
Carbon utilization	
Galactose degradation (*galM, galK, galT, galE*)	3, 3, 1, 3*
Glycogen degradation (*glgP, malQ, glgX, pgm*)	2, 2, 2, 1*
Trehalase	3
Chitinase A1	1
Bifunctional chitinase/lysozyme	2
Carbon starvation protein	4
Carbon storage	
Amylosucrase	3
Polyphosphate kinase	5
Polyphosphate glucokinase	2
Trehalose synthesis (*treS, treX, treY, treZ*)	4, 2, 3, 3
Glycogen synthesis (*glgA, glgB, glgC*)	1, 3, 3

CDS, coding sequences, * indicates a complete pathway.

The *A. agilis* Ant-EH-1 genome contains coding sequences for the degradation of a variety of carbon compounds including a complete gluconeogenesis pathway, complete pathways for galactose and glycogen degradation, chitinases (chitinase A1, and bifunctional chitinase/lysozyme) for degradation of chitin and peptidoglycan, and trehalase for trehalose degradation (Table 3). Genetic traits associated with the ability to use carbon substrates derived from the degradation of cellular material (e.g., necromass components) were found to be a major component of the community metagenome in Elephant Head, Antarctica, soils from which *A. agilis* Ant-EH-1 was isolated (Wood et al., 2024). The isolate also has coding sequences for carbon starvation protein (*cstA*) which has been shown to regulate the cAMP-CRP-dependent carbon starvation response (Schultz and Matin, 1991). Genes for trace gas metabolisms of atmospheric hydrogen, carbon monoxide, and methane were not found within the genome (Leung and Greening, 2020; Bay et al., 2021).

*A. agilis* Ant-EH-I contains genes to synthesize compounds such as trehalose, glucan, glycogen, and polyphosphates (Table 3), which can be used for carbon and energy storage to enable survival in oligotrophic conditions. Other Antarctic *Arthrobacter* species are known to store carbon as glycogen (Dsouza et al., 2015). Trehalose may additionally serve as a protectant against numerous stressors including cold and desiccation, as well as a reserve of carbon (Dsouza et al., 2015; Elbein et al., 2003; Chen et al., 2011).

## Conclusion

*Arthrobacter agilis* strain Ant-EH-1 is capable of cell division at −5 °C and has an optimum growth temperature of 25 °C. Though optimal cell division occurs at 25 °C, the highest amount of microbial respiration (activity) was measured at 5 °C. Its cold-active physiological traits were consistent with genome composition which encodes for functions related to cold adaptation including cold shock proteins, molecular chaperones, compatible solute transporters, oxidative stress response genes, and carotenoid biosynthesis genes. *A. agilis* Ant-EH-1 is also adapted for survival in oligotrophic conditions as demonstrated by genomic traits and its growth on low nutrient media (ten-fold diluted R2A). Its genome contains coding sequences for the catabolism of a variety of carbon compounds including components of cell walls from necromass. Its genome also contains genes for the synthesis of carbon and energy storage molecules such as trehalose, and polyphosphates. The ability of *A. agilis* strain Ant-EH-1 to divide and be active at −5 °C supports that the cold dry soils of Elephant Head contain viable microbial life which may be active in their environment, despite the difficulty in detecting such life in bulk soil analyses.

## Data Availability

The datasets presented in this study can be found in online repositories. The names of the repository/repositories and accession number(s) can be found at: https://www.ncbi.nlm.nih.gov/genbank/, OQ383637 https://gold.jgi.doe.gov/study?id=Gs0160646, Gs0160646.
