# Supplementary material for: Subzero cell division, respiration, and genomic traits of cryophilic Arthrobacter agilis Ant-EH-1 isolated from cold-arid Antarctic mineral soils
Source: Front Microbiol. 2025 Oct 28;16:1620620. doi: 10.3389/fmicb.2025.1620620 (PMC12602508; doi:10.3389/fmicb.2025.1620620)
Supplement: Supplementary file 2 [file Data_Sheet_1.PDF]

Supplemental Materials for: Subzero cell division, and respiration, and genomic traits in cryophilic Arthrobacter agilis Ant-EH-1 isolated from cold-arid Antarctic mineral soils by Wood et al.

1) Supplemental Methods

Phylogenetic Trees

16S rRNA gene tree

The 16S rRNA gene consensus sequence of *A. agilis* Ant-EH-1 was used to query the GenBank database using blastn (https://blast.ncbi.nlm.nih.gov/Blast.cgi) to find the closest related sequence matches. The top 50 hits based on percent identity were downloaded and an additional 8 Arthrobacter annotated as being isolated from other cryoenvironments were manually added to this list (KY386623, KY386442, KF306345, KT424968, KC986992, KR811202, JQ684255, KC442549). Multiple-sequence alignment was performed with MUSCLE (v3.8.1551) using default parameters[1]. RAxML v8.2.12 was used to construct maximum likelihood phylogenetic trees with GTRCAT model settings with 1000 bootstrapping iterations [2]. All branch support values were below 70. Trees were visualized and annotated using Interactive Tree of Life (iTOL v6.6;[3]). Where multiple sequences were found to be 100% identical, only one sequence was retained in the tree to aid visualisation.

Whole genome tree

The genome sequence data were uploaded to the Type (Strain) Genome Server (TYGS), a free bioinformatics platform available under https://tygs.dsmz.de, for a whole genome-based taxonomic analysis [4] The results were provided by the TYGS on 2025-07-07. TYGS determined closest type strain genomes by first comparing the genome of A. agilis ANT-EH-1

against all type strain genomes available in the TYGS database via the MASH algorithm and retaining the top ten matches[5]. Second, an additional set of ten closely related type strains was determined by searching the (currently) 23279 type strains available in the TYGS database for the best 50 matching type strains (according to the bitscore) based on the 16S rRNA gene. The Genome BLAST Distance Phylogeny approach (GBDP) under the algorithm 'coverage' and distance formula d5 [6] was used to determine the 10 closest type strain genomes. For the phylogenomic inference, pairwise comparisons of genomes were conducted using GBDP with 100 distance replicates calculated. Digital DDH values and confidence intervals were calculated using the recommended settings of the GGDC 4.0 [7]. The resulting intergenomic distances were used to infer a balanced minimum evolution tree with branch support via FASTME 2.1.6.1 including SPR postprocessing [8]. Branch support was inferred from 100 pseudobootstrap replicates each. The trees were rooted at the midpoint [9] and visualized with PhyD3[10]. The type-based species clustering using a 70% dDDH radius around each of the 11 type strains was done as previously described [4]. Subspecies clustering was done using a 79% dDDH threshold as previously introduced [11].

2) Supplemental Results

Ancillary stress response genes found in *A. agilis* Ant-EH-1

Oxidative stress

The *A. agilis* Ant-EH-1 genome has coding sequences for proteins that are related to protection against oxidative stress (Table 2). Oxidative stress is caused by reactive oxygen species (ROS) that are generated as by-products during normal cellular functions such as oxidative phosphorylation. ROS cause damage to cellular components including proteins, DNA,

and polyunsaturated fatty acids [12]. Oxidative stress response is required in many environments broadly but is especially crucial in sub-freezing settings. Cold temperatures increase the amount of ROS that are produced inside the cell, and oxygen becomes more soluble at cold temperatures thus increasing overall oxidative stress on cells [13–15]. *A. agilis* Ant-EH-1 has coding sequences for enzymes involved in the breakdown of ROS including superoxide dismutases, catalases, and peroxiredoxin (Table 2) [12]. *A. agilis* Ant-EH-1 also encodes a thioredoxin and thioredoxin reductase pair. Thioredoxin provides relief to oxidative stress by reducing disulfide cysteines in essential proteins to ensure proper function [16]. Thioredoxin reductases mediate thioredoxin function by keeping them reduced, allowing them to reduce other necessary proteins [17]. An encoded mycoredoxin may work in a similar way by reducing mixed disulfides formed by mycothiol [18]. Proteins exposed to reactive oxygen species are also capable of forming methionine sulfoxides via disulfide bonding [19]. *A. agilis* Ant-EH-1 encodes msrA and msrB, which are methionine sulfoxide reductases capable of returning methionine sulfoxides to their original state [19]. *A. agilis* Ant-EH-1 also contains genes involved in the synthesis of mycothiol which is the major low weight thiol in actinomycetes such as *Arthrobacter* that aid in removal of toxic peroxides [20].

DNA repair

Bacteria living in dry Antarctic surface soils are exposed to long term background radiation and cold and oxidative stress which all can damage DNA. The ability to repair DNA damage is therefore critical to cell survival on long time scales in this ecosystem. *A. agilis* Ant-EH-1 encodes the entire uvrABC system which scans DNA in search of lesions or abnormalities and excises damaged nucleotides (Table 2) [21]. Other encoded proteins that are involved in the excision repair pathway are urvD1, urvD2, and uracil-DNA glycosylase [22, 23]. *A. agilis* Ant-

EH-1 also encodes recA, recN, recO, and recF, which perform repairs on DNA damaged during replication and recombination [24–26]. A deoxyribodipyrimidein photo-lyase coding region is also present in the *A. agilis* Ant-EH-1 genome and may remove harmful cyclobutane pyrimidine dimers which form as a result of UV radiation [27]. *A. agilis* Ant-EH-1 encodes other genes (mutY, mutM, tagI, ogt) that are involved in repair of DNA that is damaged by oxidative stress, alkylating agents, and methylation to prevent transition mutations [28–30]. An encoded SOS-response regulator lexA may control many of these genes that are involved in DNA repair and stability [31].

Carotenoids, Membrane and cell wall modifications

The *A. agilis* Ant-EH-1 genome contains genes for carotenoid biosynthesis. Its ability to synthesize pigment is supported by its pink appearance when grown in the lab (Table 2). Although their mechanism of action is not well understood, carotenoids are thought to play a role in cold adaptation by regulating cell membrane fluidity [32, 33]. Increased carotenoid production associated has been associated with increased resistance to freeze-thaw stress, as identified in Arthrobacter strains closely related to A. agilis ANT-EH-1 such as A. agilis DSM 20550 (Figure S3) [34]. Carotenoids also protect against solar radiation by quenching reactive oxygen species which may be generated by UV light exposure [35]. Protection against solar radiation would be important in surface dry permafrost soils in Antarctica which are exposed to intense 24-hour light for large portions of the year, in an area with thin ozone [36]. Many bacteria that have been isolated from Antarctic soils are pigmented and those that are pigmented are found to be more resistant to environmental stressors including freeze-thaw cycles and solar radiation [37].

Additionally. bacteria may synthesize unsaturated fatty acids and branched chain fatty acids and reduce acyl chain length to retain membrane fluidity in cold environments [38]. *A. agilis* Ant-EH-1 contains genes that encode for 3-oxoacyl-[acyl-carrier-protein] synthase I, II and III (KAS-I, KAS-II, KAS-III) which are involved in fatty acid biosynthesis as well as NADPH-dependent stearoyl-CoA 9-desaturase (desA3) which is involved in desaturation [39, 40]. *A. agilis* Ant-EH-1 also encodes for hydroperoxy fatty acid reductase (gpx1) which is essential for removal of lipid hydroperoxides which disturb membrane structure due to their polarity and can participate in redox reactions [41, 42].

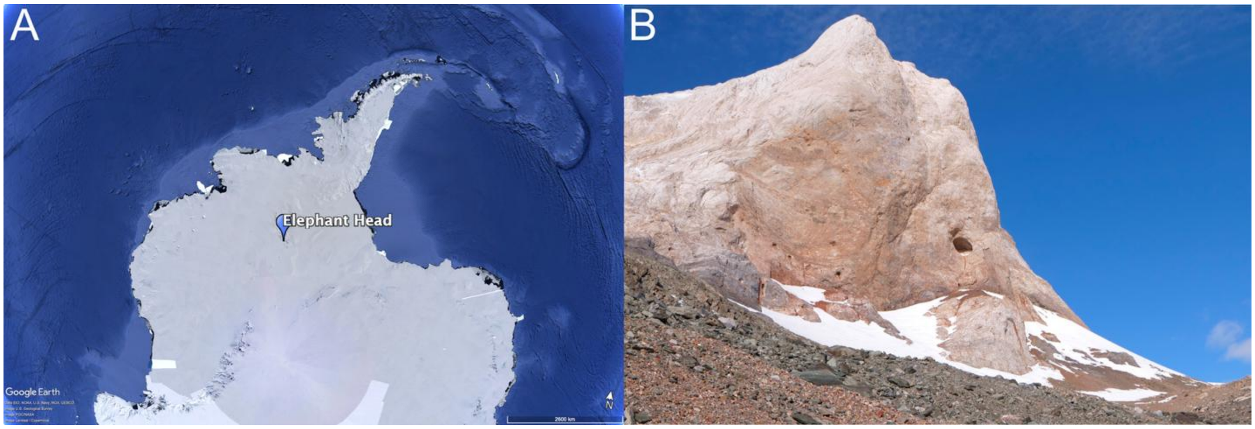

Figure S1: (A) Map of Elephant Head, Ellsworth Land, Antarctica (79°49.106'S 83°18.139W) generated using Google Maps (2025). (B) Photo of the Elephant Head site from which *A. agilis* Ant-EH-1 was isolated.

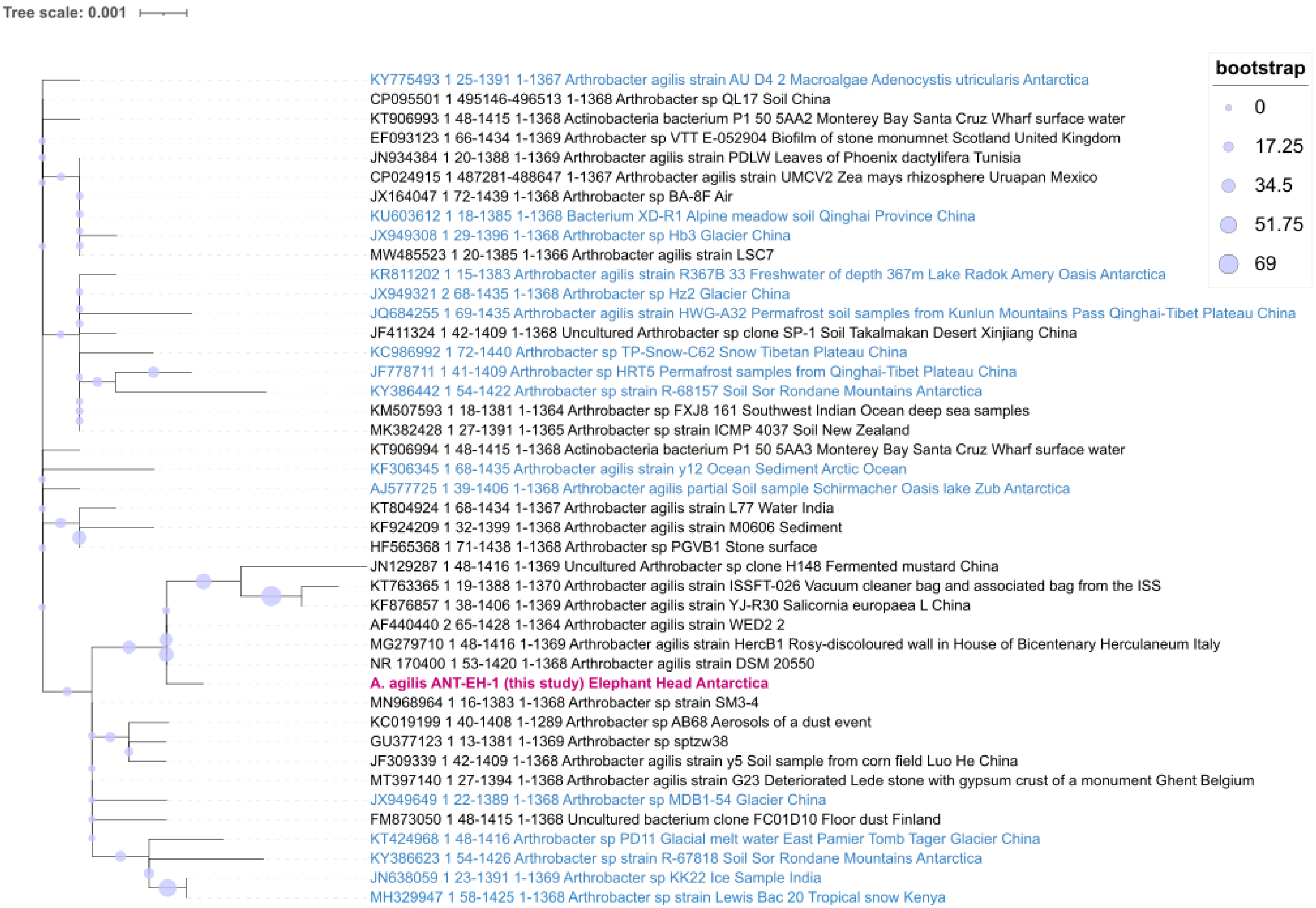

Figure S2: Phylogenetic tree (1000 bootstrap replications) of the 16S rRNA gene of *Arthrobacter agilis* strain Ant-EH-1 and close relatives. Bootstrap support is indicated by bubbles overlaying tree nodes. Organisms annotated as being from other cold environments are coloured blue.

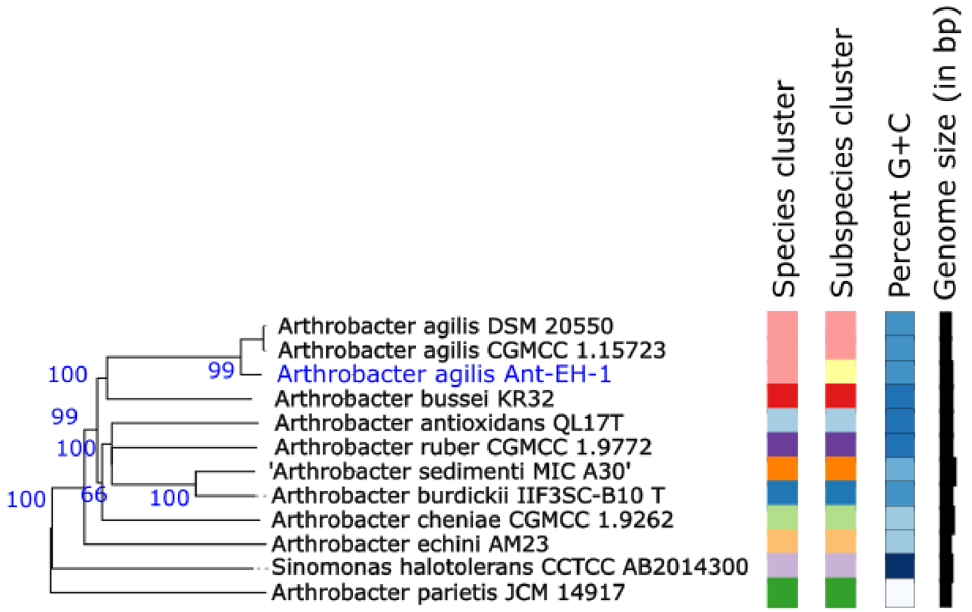

Figure S3. Whole genome phylogenetic tree of *Arthrobacter agilis* strain Ant-EH-1 and close relatives from the Type (Strain) Genome Server (TYGS). Genome BLAST Distance Phylogeny pseudo-bootstrap support values > 60 % from 100 replications, with an average branch support of 86.1 %.

Supplemental References

1. Edgar RC. MUSCLE: multiple sequence alignment with high accuracy and high throughput. *Nucleic Acids Res* 2004;**32**:1792–1797.

2. Stamatakis A. RAxML version 8: a tool for phylogenetic analysis and post-analysis of large phylogenies. *Bioinformatics* 2014;**30**:1312–1313.

3. Letunic I, Bork P. Interactive Tree Of Life (iTOL) v5: an online tool for phylogenetic tree display and annotation. *Nucleic Acids Res* 2021;**49**:W293–W296.

4. Meier-Kolthoff JP, Göker M. TYGS is an automated high-throughput platform for state-of-the-art genome-based taxonomy. *Nat Commun* 2019;**10**:2182.

5. Ondov BD et al. Mash: fast genome and metagenome distance estimation using MinHash. *Genome Biol* 2016;**17**:132.

6. Meier-Kolthoff JP et al. Genome sequence-based species delimitation with confidence intervals and improved distance functions. *BMC Bioinformatics* 2013;**14**:60.

7. Meier-Kolthoff JP et al. TYGS and LPSN: a database tandem for fast and reliable genome-based classification and nomenclature of prokaryotes. *Nucleic Acids Res* 2022;**50**:D801–D807.

8. Lefort V, Desper R, Gascuel O. FastME 2.0: a comprehensive, accurate, and fast distance-based phylogeny inference program. *Mol Biol Evol* 2015;**32**:2798–2800.

9. Farris JS. Estimating phylogenetic trees from distance matrices. *Am Nat* 1972;**106**:645–668.

10. Kreft Ł et al. PhyD3: a phylogenetic tree viewer with extended phyloXML support for functional genomics data visualization. *Bioinformatics* 2017;**33**:2946–2947.

11. Meier-Kolthoff JP et al. Complete genome sequence of DSM 30083T, the type strain (U5/41T) of Escherichia coli, and a proposal for delineating subspecies in microbial taxonomy. *Stand Genomic Sci* 2014;**9**:2.

12. Lushchak VI. Oxidative Stress and Mechanisms of Protection Against It in Bacteria. *Biochemistry (Moscow)* 2001;**66**:476–489.

13. Fasnacht M, Polacek N. Oxidative Stress in Bacteria and the Central Dogma of Molecular Biology. *Front Mol Biosci* 2021;**8**:392. https://doi.org/10.3389/fmolb.2021.671037

14. Tribelli P, López N. Reporting Key Features in Cold-Adapted Bacteria. *Life* 2018;**8**:8. https://doi.org/10.3390/life8010008

15. Chattopadhyay MK et al. Increase in Oxidative Stress at Low Temperature in an Antarctic Bacterium. *Curr Microbiol* 2011;**62**:544–546. https://doi.org/10.1007/s00284-010-9742-y

16. Olson AL et al. Solution structures of *Mycobacterium tuberculosis* thioredoxin C and models of intact thioredoxin system suggest new approaches to inhibitor and drug design. *Proteins: Structure, Function, and Bioinformatics* 2013;**81**:675–689. https://doi.org/10.1002/prot.24228

17. Mustacich D, Powis G. Thioredoxin reductase. *Biochem J* 2000;**346**:1–8.

18. Van Laer K et al. Mycoredoxin-1 is one of the missing links in the oxidative stress defence mechanism of Mycobacteria. *Mol Microbiol* 2012;**86**:787–804. https://doi.org/10.1111/mmi.12030

19. Lee WL et al. *Mycobacterium tuberculosis* expresses methionine sulphoxide reductases A and B that protect from killing by nitrite and hypochlorite. *Mol Microbiol* 2009;**71**:583–593. https://doi.org/10.1111/j.1365-2958.2008.06548.x

20. Koledin T, Newton G, Fahey R. Identification of the mycothiol synthase gene (mshD) encoding the acetyltransferase producing mycothiol in actinomycetes. *Arch Microbiol* 2002;**178**:331–337. https://doi.org/10.1007/s00203-002-0462-y

21. Orren DK, Sancar A. The (A)BC excinuclease of *Escherichia coli* has only the UvrB and UvrC subunits in the incision complex. *PNAS* 1989;**86**:5237–5241. https://doi.org/10.1073/pnas.86.14.5237

22. Williams A et al. UvrD2 is essential in *Mycobacterium tuberculosis*, but its helicase activity is not required. *J Bacteriol* 2011;**193**:4487–4494. https://doi.org/10.1128/JB.00302-11

23. Schormann N et al. Poxvirus uracil-DNA glycosylase—An unusual member of the family I uracil-DNA glycosylases. *Protein Science* 2016;**25**:2113–2131. https://doi.org/10.1002/pro.3058

24. Hu S et al. Dynamics and Cell-Type specificity of the DNA double-strand break repair protein RecN in the developmental Cyanobacterium *Anabaena* sp. strain PCC 7120. *PLoS One* 2015;**10**:e0139362. https://doi.org/10.1371/journal.pone.0139362

25. Torres R et al. *Bacillus subtilis* DisA regulates RecA-mediated DNA strand exchange. *Nucleic Acids Res* 2019;**47**:5141–5154. https://doi.org/10.1093/nar/gkz219

26. Courcelle J, Crowley DJ, Hanawalt PC. Recovery of DNA replication in UV-irradiated *Escherichia coli* requires both excision repair and RecF protein function. *J Bacteriol* 1999;**181**:916–922. https://doi.org/10.1128/JB.181.3.916-922.1999

27. Sancar A, Rupert CS. Cloning of the phr gene and amplification of photolyase in Escherichia coli. *Gene* 1978;**4**:295–308. https://doi.org/10.1016/0378-1119(78)90047-1

28. Michaels ML et al. A Repair System for 8-Oxo-7,8-dihydrodeoxyguaninet. *Biochemistry* 1992;**31**:10964–10968.

29. Zhu X et al. A model for 3-methyladenine recognition by 3-methyladenine DNA glycosylase I (TAG) from *Staphylococcus aureus*. *Acta Crystallogr Sect F Struct Biol Cryst Commun* 2012;**68**:610–615. https://doi.org/10.1107/S1744309112016363

30. Morita R et al. An O6-methylguanine-DNA Methyltransferase-like protein from *Thermus thermophilus* interacts with a nucleotide excision repair protein. *J Biochem* 2008;**144**:267–277. https://doi.org/10.1093/jb/mvn065

31. Harmon FG, Rehraur WM, Kowalczykowski SC. Interaction of *Escherichia coli* RecA protein with LexA repressor. *J Biol Chem* 1996;**271**:23874–23883.

32. Chattopadhyay MK. Mechanisms of bacterial adaptation to low temperatures. *J Biosci* 2006;**31**:157–165.

33. Jagannadham M V. et al. Carotenoids of an Antarctic psychrotolerant bacterium, Sphingobacterium antarcticus, and a mesophilic bacterium, Sphingobacterium multivorum. *Arch Microbiol* 2000;**173**:418–424. https://doi.org/10.1007/s002030000163

34. Flegler A, Lipski A. The C50 carotenoid bacterioruberin regulates membrane fluidity in pink-pigmented Arthrobacter species. *Arch Microbiol* 2021;**204**:70. https://doi.org/10.1007/s00203-021-02719-3

35. Cockell CS, Knowland J. Ultraviolet radiation screening compounds. *Biol Rev* 1999;**74**:311–345.

36. Cordero RR et al. Persistent extreme ultraviolet irradiance in Antarctica despite the ozone recovery onset. *Sci Rep* 2022;**12**:1266. https://doi.org/10.1038/s41598-022-05449-8

37. Dieser M, Greenwood M, Foreman CM. Carotenoid Pigmentation in Antarctic Heterotrophic Bacteria as a Strategy to Withstand Environmental Stresses. *Arct Antarct Alp Res* 2010;**42**:396–405. https://doi.org/10.1657/1938-4246-42.4.396

38. Chattopadhyay MK, Jagannadham M V. Maintenance of membrane Fluidity in Antarctic bacteria. *Polar Biol* 2001;**24**:386–388. https://doi.org/10.1007/s003000100232

39. Chang Y, Fox BG. Identification of Rv3230c as the NADPH oxidoreductase of a two-protein DesA3 acyl-CoA desaturase in *Mycobacterium tuberculosis* H37Rv. *Biochemistry* 2006;**45**:13476–86. https://doi.org/10.1021/bi0615285

40. Phetsuksiri B et al. Unique mechanism of action of the thiourea drug isoxyl on *Mycobacterium tuberculosis*. *J Biol Chem* 2003;**278**:53123–30. https://doi.org/10.1074/jbc.M311209200

41. Gaber A et al. Induction and functional analysis of two reduced nicotinamide adenine dinucleotide phosphate-dependent glutathione peroxidase-like proteins in Synechocystis PCC 6803 during the progression of oxidative stress. *Plant Physiol* 2004;**136**:2855–2861. https://doi.org/10.1104/PP.104.044842

42. Girotti AW. Lipid hydroperoxide generation, turnover, and effector action in biological systems. *J Lipid Res* 1998;**39**:1529–1542. https://doi.org/10.1016/S0022-2275(20)32182-9
